# A Complex Left Internal Mammary Artery Intervention

**DOI:** 10.7759/cureus.40593

**Published:** 2023-06-18

**Authors:** Mansoor Ahmad, Kailash Pant, Mena Henien, John Rashid

**Affiliations:** 1 Cardiology, University of Illinois College of Medicine Peoria, Peoria, USA; 2 Internal Medicine, University of Illinois College of Medicine Peoria, Peoria, USA; 3 Interventional Cardiology, University of Illinois College of Medicine Peoria, Peoria, USA

**Keywords:** complex percutaneous coronary intervention, coronary artery by-pass grafting, chronic stable angina, coronary artery intervention, left internal mammary artery

## Abstract

There is a variety of conduits utilized as vascular grafts in coronary artery bypass grafting (CABG). Post-CABG graft rate of failure varies depending on the type of conduit used, with the highest failure rates seen in saphenous vein grafts (SVG). Patency rates of SVG are reported to be about 75% at 12-18 months. Left internal mammary artery (LIMA) grafts have shown higher long-term patency rates when compared to other arterial and venous grafts; however, LIMA occlusions occur, most commonly in the early postoperative period.

Percutaneous coronary intervention (PCI) of LIMA graft can be challenging based on the location, the length of the lesion, as well as other factors such as vessel tortuosity. Here we present a case of a complex intervention for osteal and proximal LIMA chronic total occlusion (CTO) in a symptomatic patient. Long stent delivery is usually a challenge in LIMA intervention; however, it was successfully achieved here by placing two overlapping stents. This intervention was also complicated by the tortuosity of the lesion, as well as the difficult cannulation of the left subclavian artery requiring a longer sheath for guide support.

## Introduction

Left internal mammary artery (LIMA) is frequently used as the vascular graft to bypass coronary arteries for severe coronary artery disease. Although LIMA grafts have shown higher long-term patency rates when compared to other arterial and venous grafts, LIMA occlusion does occur, most commonly in the early postoperative period. Atherosclerotic occlusion of LIMA is not very common, but when present, it's usually a chronic process. Percutaneous coronary intervention (PCI) of LIMA graft can be challenging based on the location, and the length of the lesion, as well as other factors such as vessel tortuosity. We present the case of a complex LIMA intervention [1,2].

## Case presentation

An 81-year-old patient with a past medical history of hypertension, hyperlipidemia, coronary artery disease, and coronary artery bypass surgery (dated 10/18/2011), with LIMA grafted to left anterior descending artery (LAD), saphenous vein graft (SVG) grafted to distal right coronary artery (RCA), and SVG graft to obtuse marginal 2 (OM2; this last graft was known to be occluded). His medications included aspirin 81 mg, rosuvastatin 10 mg, amlodipine 10 mg, and nitroglycerin 0.4 mg tablets as needed for chest pain.

**Figure 1:**
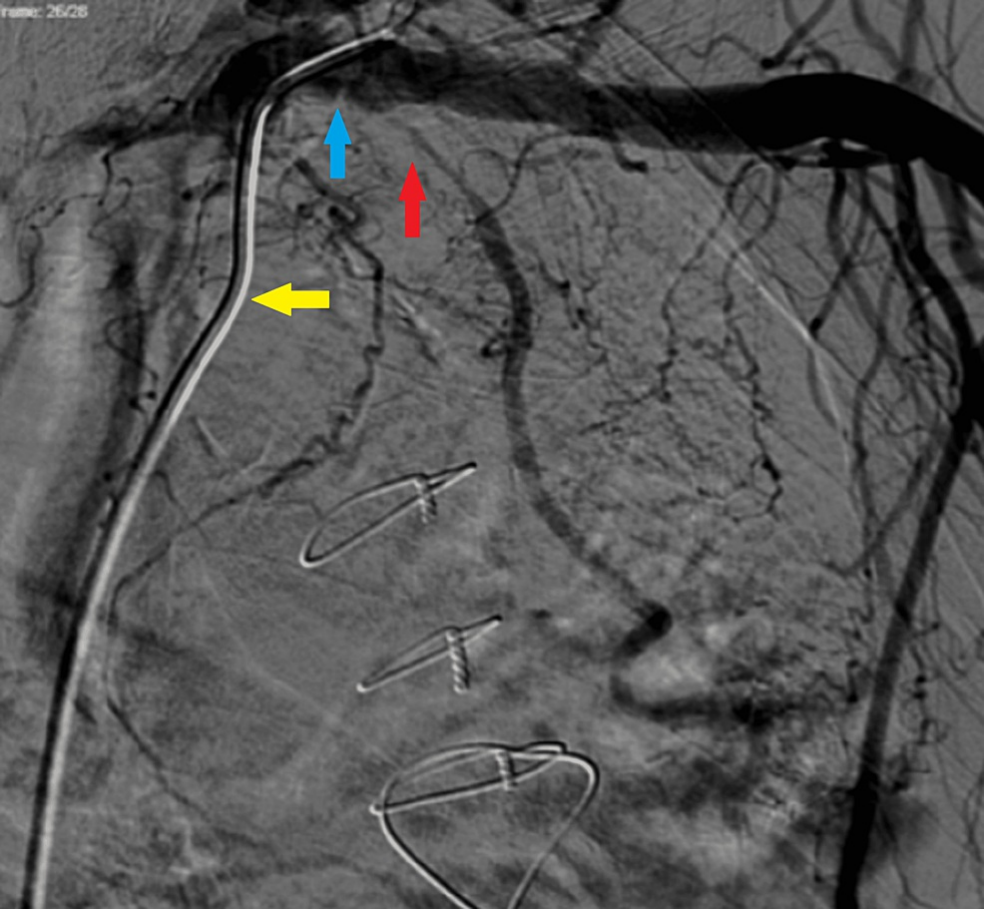
LIMA stenosis involving ostial and proximal segments LIMA: Left Internal Mammary Artery Digital subtraction image. The guide catheter (yellow arrow) is engaged in the proximal left subclavian artery (blue arrow), showing severe stenosis of LIMA (red arrow).

The patient presented to the hospital on 01/05/2021 with a chief complaint of typical chest pain associated with one syncopal episode. He also endorsed a one-month history of orthopnea and dyspnea on exertion. Initial labs were significant for elevated troponin and B-type natriuretic peptide (BNP). Chest radiograph showed left loculated pleural effusion. EKG demonstrated ST-segment elevation in aVR, and depression in leads I, aVL, and V2-V6. Transthoracic echocardiogram showed an ejection fraction of 30%, abnormal septal wall motion, with hypokinesis in mid to apical antero-septum/anterior wall, inferior, anteroseptal walls, and apical cap. The patient was started on a heparin infusion. A left heart catheterization revealed the culprit lesion to be ostial/proximal LIMA to LAD with 100% chronic total occlusion (TIMI 0 flow) (Figure 1).

Since LAD was extensively calcified with 95% ostial proximal stenosis, the option of intervening on LAD was not considered. The lesion in LIMA was calcified and extended for 30 millimeters. It also showed severe tortuosity, with unfavorable total occlusion and collateralized by axillary artery, giving competitive flow to LAD which had some native flow with 95% proximal LAD stenosis.

**Figure 2:**
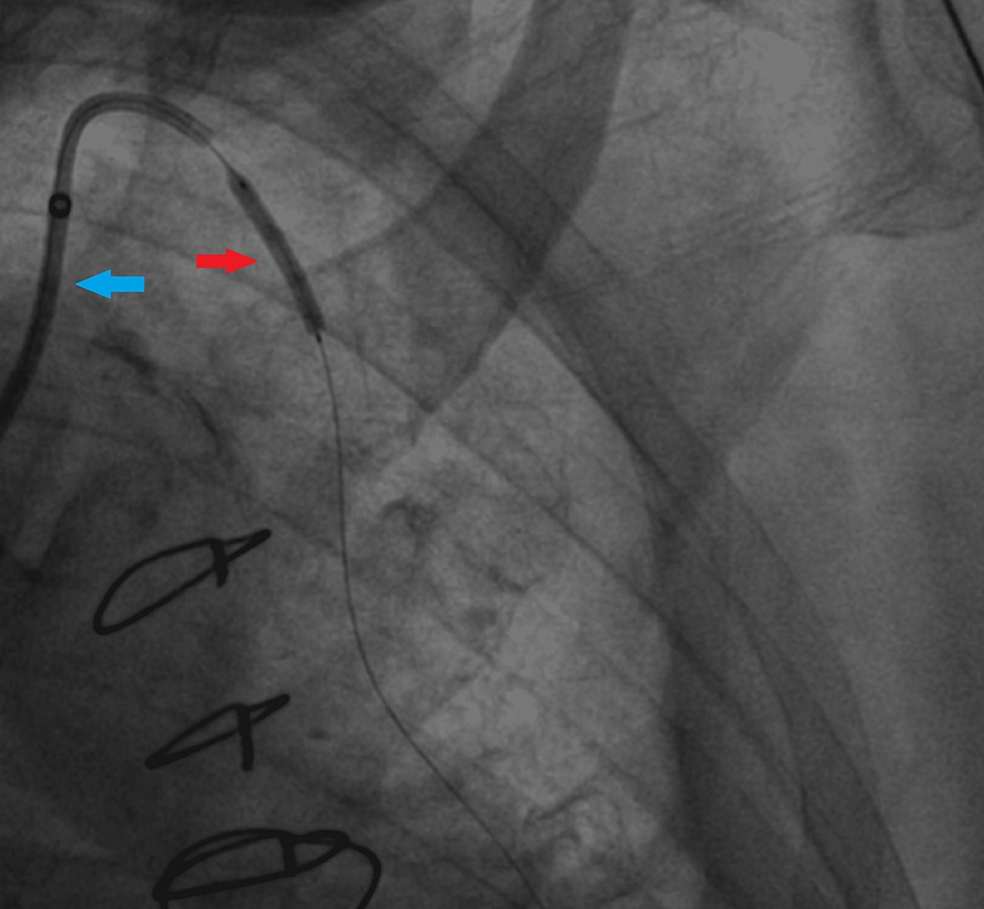
Placement of the first stent in ostial LIMA with balloon dilation LIMA: Left Internal Mammary Artery A long sheath (blue arrow) can be seen with balloon dilation to deploy the first stent (red arrow).

**Figure 3:**
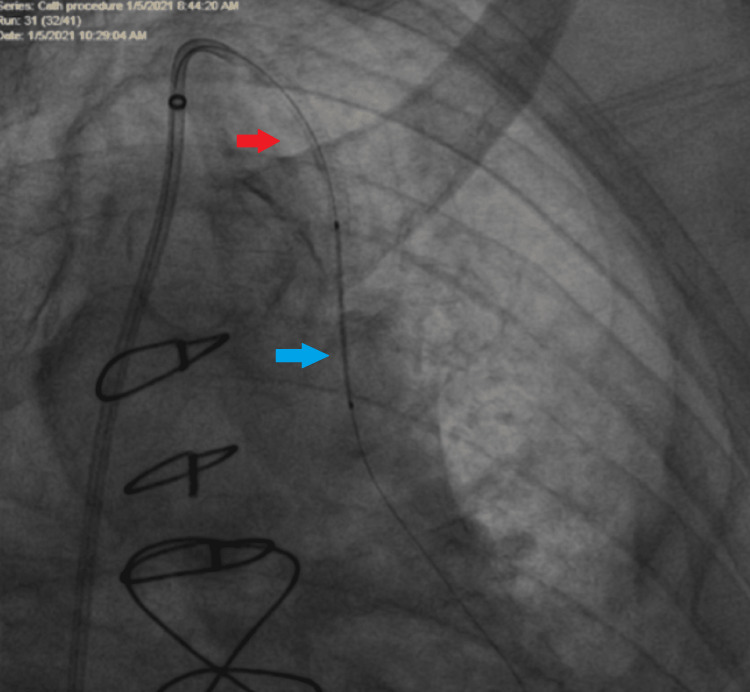
Delivery of the second stent in the proximal LIMA LIMA: Left Internal Mammary Artery Delivery of the second stent (blue arrow) in the proximal LIMA, the ostial stent is already well deployed (red arrow). Distal stent being delivered to proximal LIMA.

**Figure 4:**
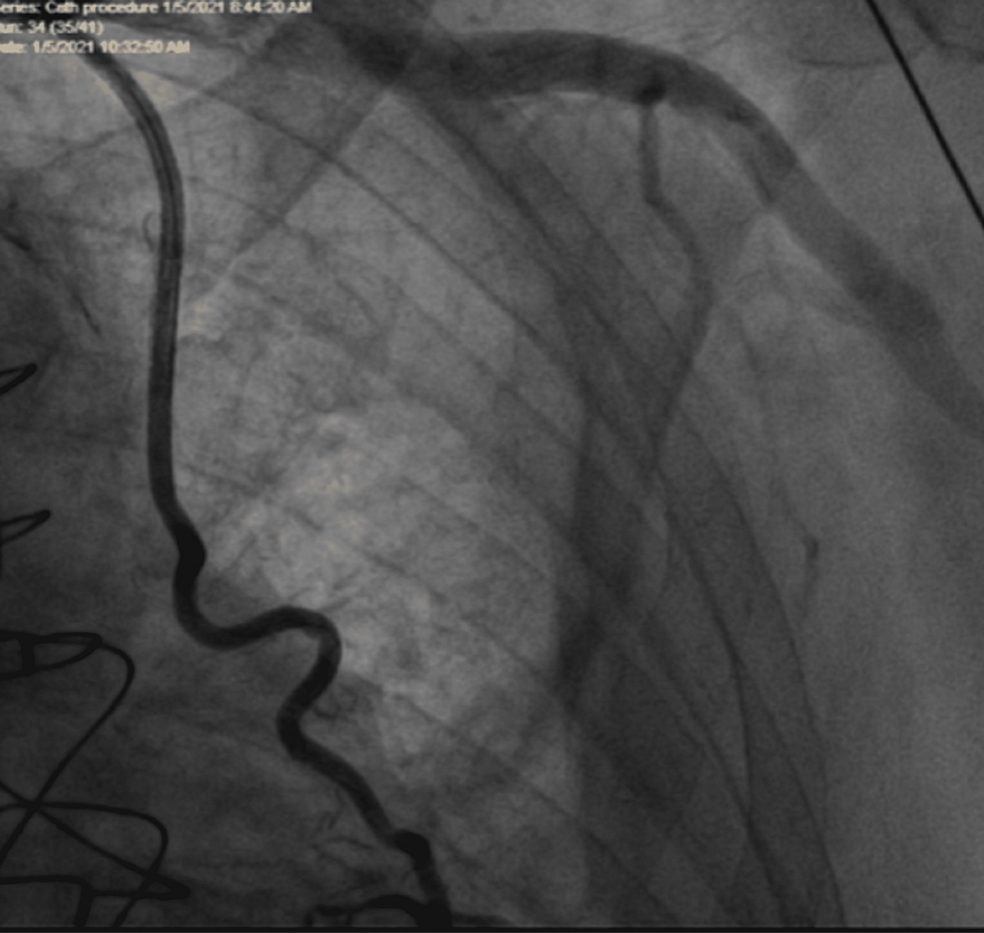
Final result of LIMA with TIMI III flow after deployment of two stents LIMA: Left Internal Mammary Artery, TIMI: Thrombolysis in Myocardial Intervention flow rate III

Access to LIMA was complex due to tortuosity of the left subclavian, and a long femoral sheath was used, followed by 5F 100cm HH 1 Impress Catheter to inject the LIMA graft. A Miracle Bro 12 wire was used to cross the lesion as the use of regular PT2 Guidewire was unsuccessful in accomplishing this task (Figure 2). Two drug-eluting stents were placed; one proximal 3 x 38 mm overlapped with a 3 x 33 mm stent distally (Figures 2-3). Post-procedure TIMI-III flow was demonstrated (Figure 4).

## Discussion

Post-CABG graft rate of failure varies depending on the type of conduit used, with the highest failure rates in saphenous vein grafts. Patency rates are known to be about 75% in SVG at 12-18 months [3,4]. LIMA to LAD grafts have been shown to be more durable when compared to other arterial and vein grafts. It has the highest patency rates of above 90% even at 10 years [5,6].

Here we present a case of a complex intervention for ostial and proximal LIMA chronic total occlusion (collateralized by axillary branches) in a symptomatic patient. The use of a long sheath for reaching the left subclavian complicates this case as maneuvering the equipment through the long sheath is very challenging, especially to cannulate LIMA. The use of Miracle Bro 12 wire is not common but was required considering the stiffness of this lesion. Long stent delivery is usually a challenge in LIMA intervention, especially for ostial and proximal lesions, due to tortuosity of the vessels such as in this patient. In addition, the origin of LIMA at the subclavian is close to 90°, which adds a challenge to the delivery of a longer stent in this ostial lesion; however, it was successfully achieved here and two overlapping stents were deployed.

Consideration could have been given to the left radial approach in this patient as well; however, considering the complexity and location of the lesion, the guide support was better through the femoral approach which was adopted in this patient, however, the femoral approach required a long sheath to engage the lesion.

## Conclusions

Multiple aspects of complex intervention need to be considered during LIMA intervention. Using a long sheath can help to stabilize the delivery system. We need to familiarize ourselves with the different wires available to cross a complex lesion. A longer stent can be difficult to deploy, especially in proximal LIMA interventions. A multidisciplinary approach, with consultation with other partners, can help navigate and treat these lesions percutaneously.
